# First Nations Food Environments: Exploring the Role of Place, Income, and Social Connection

**DOI:** 10.1093/cdn/nzaa108

**Published:** 2020-07-01

**Authors:** Chantelle Richmond, Marylynn Steckley, Hannah Neufeld, Rachel Bezner Kerr, Kathi Wilson, Brian Dokis

**Affiliations:** Department of Geography, Western University, London, Ontario, Canada; Carleton University, Ottawa, Ontario, Canada; University of Waterloo, Waterloo, Ontario, Canada; Cornell University, Ithaca, NY, USA; University of Toronto–Mississauga, Mississauga, Ontario, Canada; The Southwest Ontario Aboriginal Health Access Centre, London, Ontario, Canada

**Keywords:** Indigenous health, food insecurity, food environments, urban, environmental dispossession, Indigenous food sovereignty, environmental repossession, Canada

## Abstract

**Background:**

In Canada, few studies have examined how place shapes Indigenous food environments, particularly among Indigenous people living in southern regions of Ontario.

**Objective:**

This paper examines and compares circumstances of food insecurity that impact food access and dietary quality between reserve-based and urban-based Indigenous peoples in southwestern Ontario.

**Methods:**

This study used a community-based survey containing a culturally adapted food-frequency questionnaire and cross-sectional study design to measure food insecurity, food access, and dietary quality among Indigenous respondents living in urban (*n* = 130) and reserve-based (*n* = 99) contexts in southwestern Ontario.

**Results:**

Rates of food insecurity are high in both geographies (55% and 35% among urban- and reserve-based respondents, respectively). Urban-based participants were 6 times more likely than those living on-reserve to report 3 different measures of food insecurity. Urban respondents reported income to be a significant barrier to food access, while for reserve-based respondents, time was the most pressing barrier. Compared with recommendations from Canada's Food Guide, our data revealed overwhelming trends of insufficient consumption in 3 food categories among all respondents. Close to half (54% and 52%) of the urban- and reserve-based samples reported that they eat traditional foods at least once a week, and respondents from both groups (76% of urban- and 52% of reserve-based respondents) expressed interest in consuming traditional foods more often.

**Conclusions:**

Indigenous Food Sovereignty and community-led research are key pathways to acknowledge and remedy Indigenous food insecurity. Policies, social movements, and research agendas that aim to improve Indigenous food security must be governed and defined by Indigenous people themselves. Indigenous food environments constitute political, social, and cultural dimensions that are infinitely place based.

## Introduction

Indigenous food insecurity is a significant and persistent matter in Canada, one that deserves both greater research attention and policy action (1–3). Over the past few decades, scholars have shed light on the connections between nutrition transition and Indigenous dietary patterns (4–6); the increasing prevalence of diet-related diseases such as obesity, type 2 diabetes, and heart disease (7–11); and the links between these diet-related diseases and a range of social determinants of health (12–14). To date, however, very few studies have examined how features of place shape Indigenous food environments—that is, how does one's physical and social surroundings influence food choice, access, and availability? While it is now well understood that Indigenous conceptualizations and experiences of health are fundamentally shaped by place, and connections with the land (15, 16), the ways in which place shapes Indigenous dietary patterns and food security remain relatively understudied, particularly in urban areas. In addressing this gap in knowledge, our research is guided by the following question: how does place impact the differential food security experiences and dietary patterns of urban- and reserve-based Indigenous people in southwestern Ontario? Informed by a long-term, community-based research project, our work draws from structured surveys (*n* = 229) with First Nations people who live in the city of London, Ontario, Canada, and on a nearby First Nation reserve, to examine and describe the place-based experiences of Indigenous food security. Herein, we pay particular attention to how place impacts experiences of food environments, dietary practices, and perceptions of food insecurity in both contexts.

### Theoretical background: connecting place and Indigenous food security

In Canada, Indigenous populations experience disproportionate levels of both severe (acute) and chronic food insecurity (1). The concept of food security has received much theoretical and applied debate and interpretation over its use and meaning (17–19). At the World Food Summit in 1996 (20), a definition was agreed upon by member states: “food security exists when all people, at all times, have physical and economic access to sufficient, safe and nutritious food to meet their dietary needs and food preferences for an active and healthy life.” At a broad level, food security implies sufficient access, availability, and use of nutritious and culturally preferable foods. Populations with poor access to the social determinants of health—including high levels of food insecurity—are overrepresented by obesity, chronic disease, and other diet- and lifestyle-related noncommunicable conditions (21). In northern and remote contexts of Canada, patterns of food insecurity are linked to a number of important changes, including the following: declining access to healthy and affordable foods, shifts to the wage economy, climate change and other environmental changes, and the erosion of Indigenous knowledge (22–25).

By comparison, southern Indigenous food environments have received significantly less attention (26, 27), despite the fact that over the past decade there has been steady growth of First Nations people in southern Canadian cities. One significant trend among Indigenous populations in southern Ontario, and more broadly in southern Canada, is urbanization. At the national level, the proportion of urban Indigenous populations increased from 13% to 53% between 1961 and 2006 (28). Provincially, Ontario has experienced the most dramatic rates of urbanization. In 2018, 63% of Indigenous people in Ontario lived off-reserve (29). Urbanization has been accompanied by a nutrition transition characterized by decreased dietary diversity, declining access to traditional foods, and the increased tendency towards “industrial diets,” the core pillars of which are fats, sugars, salts, and processed foods. Rapid dietary change has had perilous health consequences in many global contexts, but Indigenous groups face disproportionate adverse health effects, including higher rates of obesity, cardiovascular disease, hypertension, anemia, and type 2 diabetes (1, 4, 7). Indigenous people living off-reserve are also more than twice as likely to experience hunger and food insecurity compared with non-Indigenous Canadians; 27% of Indigenous Canadians are food insecure compared with 11% of non-Indigenous households (30).

In large measure, research exploring the relationships between food and the social determinants of health in Indigenous contexts has been written largely from the vantage point of public health, social epidemiology, and nutrition scholars. Notably, while features of place and geographic location have played an important role in the ways this body of research has been both organized and presented, there has been little theoretical engagement with “place” as a feature that shapes food environments in measurable ways. Geography offers a disciplinary context that can contribute to improved understanding of the connections between people, food, and place.

In the broader discipline of human and social geography, an important theoretical development relates to a relatively new subdiscipline of critical human geography: the geographies of Indigenous health. This emerging area of study is informed most directly by the fields of health geography and Indigenous geography, with research problems that center foundationally around the complex and changing relationship between Indigenous people's health and the environment (31). As noted by Richmond and Big-Canoe [(31), p. 179], key questions asked by researchers in this discipline include the following: How is the health and well-being of Indigenous peoples and communities shaped by features of the environments within which they live? What processes work to affect the health and environments of Indigenous peoples, and how so? Can Indigenous engagement in research support positive change for Indigenous well-being and environmental protection? In response to calls for research that serves the self-determining goals of Indigenous communities (32, 33), the geographies of Indigenous health support a methodological imperative that places Indigenous communities and their concerns at the forefront of research about Indigenous health and the environment, including food environments (34, 35).

The geographic literature on food environments offers an important lens through which to understand the differential Indigenous experiences and social perceptions of food environments and food security on- and off-reserve. Food environments play a meaningful role in shaping inequalities in such things as dietary practices, food access, obesity prevalence, and hunger (36, 37), in addition to how broader interconnections between socioeconomic, cultural, environmental, political-economic structures, and historical processes work together to impact household food security; place remains key to people's health and diets (38). The literature on food environments emphasizes how dynamic relationships between low income and exposure to low-quality food environments undermine food security and heighten risk to diet-related diseases. For example, in North America, low-income neighborhoods tend to have fewer grocery stores, less readily available fresh healthy foods, more convenience stores, and higher food prices than middle-income neighborhoods, increasing residents’ vulnerability to develop diet-related conditions such as obesity (39, 40).

Our research contributes to this literature by shedding light on how place impacts food choices among Indigenous people and the differential experiences of food security for reserve- and urban-based Indigenous people in southwestern Ontario. It also contributes to the growing literature on the relationship between urbanization and Indigenous food security. In the past decade, an emerging body of research has begun to examine the consequences of urbanization on Indigenous well-being, health, and food security, but there has not yet been a systematic study of the determinants of food security between reserve-based and urban-based Indigenous peoples (41, 42). While place of residence clearly impacts Indigenous dietary practices and food security, there is little known about the differential nature of food insecurity between on-reserve and off-reserve households. Our paper builds on the existing research, both by exploring the importance of place and the ways in which it can shape Indigenous experiences of food security, but also by engaging in a community-based approach that both supports and empowers Indigenous communities and organizations to be more self-determining on matters relating to their health and well-being (31, 34).

## Methods

This research draws from a community-based, cross-sectional study that explored the social and spatial determinants of food security among 229 First Nations respondents in southwestern Ontario: 130 who lived in the city of London and 99 who lived on a First Nation reserve ∼30 km from the city center. Within the Canadian context, the province of Ontario is home to the largest share of the national Indigenous population (21%), of whom the majority are First Nations (43). Often, Indigenous populations are classified by place of residence: those who live “on-reserve” (land set aside or “reserved for Indians” by the government) and those who live “off-reserve” (either in rural or urban areas). In this paper, we use the terms “reserve-based” and “urban-based” to refer to the “on-reserve” and “off-reserve” populations, respectively. The greater London area is home to relatively large populations of both reserve-based and urban-based Indigenous populations, making this an appropriate site for exploring differential food security and food-provisioning experiences between these participant groups. In particular, the close spatial proximity of reserve-based and urban-based study participants helps limit possible variances in food insecurity as a result of geographic distance (i.e., distance from food markets, geographically distinct dietary preferences between regions).

Our research is the result of a community-based research study; the findings shared here describe results from the first phase of the Southwest Ontario Aboriginal Health Access Centre (SOAHAC) Food Choice Study. SOAHAC is a community-based organization with goals to “improve access to, and the quality of, health services for Aboriginal peoples in Southwestern Ontario in the spirit of partnership, mutual respect and sharing” (44). This study was collaboratively designed by researchers from Western University and the University of Toronto Mississauga, and staff from SOAHAC. This study grew from a shared recognition by SOAHAC's dietitians and allied health professionals that mainstream approaches to chronic disease prevention were not working. SOAHAC staff expressed frustration with an individualized health care model, and articulated that that they did not want to continue to promote “more balanced diets” or to coach individual patients on “healthy eating,” when the root causes of diet-related diseases extend far beyond the individual, and often encompass factors such as income, single-parenthood, experiences of trauma, and social isolation. There was an important acknowledgment that understanding patterns of food choice and food security required more holistic approaches that engaged with families and communities to address wider social determinants of health, especially among Indigenous women, for whom the food and health impacts of colonization and environmental dispossession have been unique (41, 42).

Our survey instrument was developed over a 6-mo period. During this time, we held a number of focus group discussions and small-group meetings with members of the wider First Nations community and SOAHAC staff to ensure the survey was culturally and locally relevant, and that its conception was guided by Indigenous perspectives on Indigenous food security. The final iteration of the survey included questions contained in 5 main sections: *1*) background information [i.e., age, gender, marital status, place of residence (on- or off-reserve location)]; *2*) food consumption and food frequency, including traditional foods; *3*) food sources; *4*) food security; and *5*) household income.

To measure the consumption frequency of both market and traditional foods, we worked with our community partners to adapt a food-frequency questionnaire (FFQ). In the first section of our adapted FFQ, respondents were asked to describe how often they consumed different types of market food and drinks. Specifically, respondents were asked the following question: In the last week, how often did you eat or drink the following?

Market food included the following 12 categories: milk and milk products; meat and eggs; fish; beans/seeds/nuts; fruits; 100% fruit juice; vegetables; grains; salty snacks, sweets; water; and soft drinks. Eight frequencies were available: ≥6 times/d, 4–5 times/d, 2–3 times/d, 1 time/d, 5–6 times/wk, 2–4 times/wk, 1 time/wk, and rarely or never.

Traditional food consumption was measured in the second section of our adapted FFQ. First, we asked respondents to provide a list of the traditional foods they eat most often. We then asked respondents to describe how often they consumed traditional foods. Specifically, respondents were asked the following question: In the last month, how often did you eat or drink the traditional foods you listed above?

Eight frequencies were available: ≥6 times/d, 4–5 times/d, 2–3 times/d, 1 time/d, 5–6 times/wk, 2–4 times/wk, 1 time/wk, 1–3 times/mo, or never. Note that, in comparison with market food frequency, which respondents were asked to recall “over the past week,” traditional food frequency was examined “over the past month.” We made this decision, as we were not confident of daily or weekly consumption of traditional foods, and thus extended the temporal framing to recall over the past month.

Respondent perceptions of food insecurity were measured through 3 questions from the SOAHAC Food Choice Study, which were informed by the Household Food Insecurity Access Scale (HFIAS) to assess a household's experiences of, and access, to food (45). Specifically, respondents were asked the following questions:

- In the last month, did you worry about not having enough food?
- In the last month, did money prevent you and your family from eating the foods you prefer?
- In the last month, did you ever go to sleep hungry?

Before any research was conducted, the overall study was presented to and approved by SOAHAC's Board of Directors. This research was approved by Western University's Non-Medical Research Ethics Board (protocol #15543S).

To initiate data collection and introduce community members to the study, 2 large feasts were held, one in each study site. The study team, including SOAHAC staff, agreed that this was a culturally appropriate way to introduce the project, as community feasts traditionally serve as a space to share food and strengthen relationships. During the feasts, community members were invited to participate in the self-administered survey. Two additional rounds of recruitment occurred at local SOAHAC offices to involve clients who could not attend the feasts. This recruitment process was chosen with the recognition that the perceptions and experiences of SOAHAC clients offer an important window on a demographic group highly vulnerable to food insecurity.

In our analysis, descriptive statistics were used to establish commonalities of and differences between reserve-based and urban-based Indigenous participants in 3 categories: food security, food access, and food frequency. Data analysis was completed using Microsoft Excel software. Percentages and frequencies were calculated to summarize participant food security and to evaluate differences in consumption of different food groups by place of residence. Participants were considered food insecure if they responded positively to ≥1 of the food security baseline questions from the survey. Chi-square tests (*P* > 0.05) were used to test differences in levels of food insecurity.

## Results

### Place and Indigenous experiences of food insecurity

Our study sample was overrepresented by low-income individuals; 74% of respondents reported their household income to be <$30,000. The study sample was also overrepresented by women (73%: 75% on-reserve, 72% off-reserve) and by households led by single mothers (49%: 54% off-reserve, 43% on-reserve). The composition of our study sample reflects the particular vulnerability of low-income Indigenous women in the greater London area, a trend consistent with regional studies on the gendered nature of food security (46). The high number of women and the low number of male participants in this study meant that we were unable to conduct an in-depth analysis of gender-based differences. Further, our comparison of survey responses between men and women revealed there were no significant gendered differences in food insecurity. Thus, the remainder of our results focus on the place-based nature of food insecurity.

### Food insecurity

The results of our survey illustrate that both reserve- and urban-based respondents experience significantly higher levels of food insecurity in comparison with national levels. In concrete terms, 35% of reserve-based and 55% of urban-based respondents describe themselves as food insecure, compared with 8.3% of Canadians (30). At the same time, there are important spatial differences between these groups. Not only was perceived food insecurity disproportionately greater among urban individuals (*P* = 0.00053, χ2 = 7.788, *df* = 1), the disparity was even more stark when comparing spatial differences in high levels of food insecurity—that is, when participants responded positively to all 3 of the HFIAS questions. Urban-based participants were 6 times more likely than those living on-reserve to report that in the past month they had gone to bed hungry, worried about not having enough food for their families, and had been unable to purchase preferred foods because of money (*P* = 0.0080, χ^2^ = 7.044, *df* = 1). The results are clear: urban First Nations respondents are far more likely to be food insecure. For example:

- 18% of urban and 2% of reserve-based First Nations participants reported that they went to bed hungry because they did not have enough food to eat;
- 41% of urban-based and 29% of reserve-based respondents described that they worry about having enough food to eat; and
- 41% of urban based and 5% of reserve-based First Nations indicate that their family is unable to eat preferred foods because of a lack of money.

### Food access

Both reserve- and urban-based respondents identified key barriers to accessing foods that they prefer, including time (both time for food provisioning and food preparation), money, and distance. However, there were significant spatial differences between groups with respect to the dominant barrier to eating preferred foods. For example, 58% of reserve-based respondents and only 24% of urban participants indicated time as the most significant constraint to eating preferred foods (*P* < .0001, χ^2^ = 24.858, *df*  = 1). Additionally, 20% of reserve-based respondents and 39% of urban participants described money as a major barrier (*P* = 0.00045, χ^2^ = 8.076, *df* = 1). Over 20% of both population groups reported that distance and lack of access to transportation made it difficult to access a grocery store.

Accessing healthy foods was difficult for all study participants, and there were no significant differences between the primary food source location for urban- and reserve-based participants. Both groups relied primarily on grocery stores, with 98% of both populations reporting shopping at grocery stores every month and 60% of both populations reporting shopping at convenience stores at least once a month. Many participants also described making very frequent food purchases from convenience stores, with ∼27% of urban-based and 29% of reserve-based residents buying food from convenience stores on a daily or weekly basis.

Despite similar patterns in the dominant barriers to food security, there was significant variance in the relative importance of secondary food sources (i.e., gardens, soup kitchens, community events) between urban- and reserve-based groups. For example, reserve-based participants were significantly more likely to source food through gardening (*P* = 0.0045, χ^2^ = 15.039), with 54% of reserve-based participants utilizing home gardens as a food source in the previous month compared with only 45% of urban-based participants. In addition, there was significant variation in the use of soup kitchens, food banks, and church meals by urban-based and reserve-based participants; urban participants relied much more heavily on soup kitchens (33%) than reserve-based participants (11%). In comparison with the broader Canadian population, both groups were disproportionately dependent on these food sources. At the national level, ∼2.5% of Canadians make use of food banks monthly (47), while in our study 44% of urban-based and 17% of reserve-based participants reported depending on food banks or church meals >3 times/mo (*P* = 0.0013, χ^2^ = 17.899).

### Dietary quality

At the time of this study, *Eating Well with Canada's Food Guide* food-based recommendations revolved around the 4 food groups: meat and alternatives, milk and alternatives, vegetables and fruit, and grain products (48). [Canada’s Food Guide was updated in early 2019 based on an evidence review and public consultations. It moves away from the categorization model of food-based guidance to include more holistic recommendations on healthy food choices and eating habits (reference new food guide: https://food-guide.canada.ca/en/).  Health Canada and Indigenous Services Canada are continuing to work with Indigenous people to support the development of an updated set of guidance tools.] Participants’ responses to the adapted FFQ that compiled data on the daily consumption of the 4 food categories and monthly consumption of traditional foods such as wild meats, berries, wild rice, and other prepared foods such as bannock and corn soup were compared with the range of recommended servings for men and women aged 19–50 y. Overall, there were no significant differences between the self-reported dietary intake patterns of urban- and reserve-based respondents compared with these national recommendations (see **Table 1**). However, in the meat and alternatives food category, urban participants were more likely to consume too much meat (30% compared with 20%), while a disproportionate number of reserve-based participants reported not consuming enough meat and alternatives (43% compared with 19% of urban dwellers). Despite the relative similarities in the other 3 food categories, it is important to emphasize that the dietary patterns of both groups do not adhere to the guide's food-based recommendations (see Table 1). Our data revealed overwhelming trends of insufficient consumption in 3 food categories. For example, consumption of milk and alternatives was lower overall, with only 34% of reserve-based and 27% of urban-based participants consuming the recommended range of servings. Fruit and vegetable intakes were also lower, with 27% of reserve-based and 32% of urban-based diets adhering to recommendations in this category. Grain product consumption was also extremely low; only 3% of reserve-based and 5% of urban-based participants reported consuming the recommended range of between 6 and 8 servings daily.

**TABLE 1:** Comparing reserve-based and urban-based Indigenous food consumption patterns with Canada's Food Guide daily recommended serving ranges for men and women

	Canada's Food Guide,^1^ servings/d	On-reserve (*n* = 99),^2^ % [*n*]	Urban-based (*n* = 130),^2^ % [*n*]
Vegetables and fruits	7–10	27 [26]	32 [42]
Grain products	6–8	3 [3]	5 [6]
Milk and alternatives	2	34 [34]	27 [35]
Meat and alternatives	2–3	50 [54]	37 [48]

1 The range of number of recommended servings indicated is for men and women aged 19–50 y since recommended servings are slightly higher for men in all food categories except for milk and alternatives.

2 Percentages indicate proportion of sample who meet recommended daily servings, followed by the number of respondents in brackets.

Alongside data indicating nutritional deficits in 3 of the 4 food categories, our research illustrates an overreliance on salty snacks, sweets, and soft drinks, with overreliance determined by consumption of the item ≥1 times daily. Over 25% of urban- and 34% of reserve-based participants reported consuming high amounts of salty snacks. Thirty-four percent of urban-based and 37% of reserve-based respondents consumed excessive sweets. And 54% of urban- and 40% of reserve-based participants consumed high amounts of soft drinks.

With regard to traditional food consumption, 54% and 52% of the urban- and reserve-based samples reported that they eat traditional foods at least once a week. One-quarter (25%) of the reserve-based sample reported eating traditional foods 2–4 times/wk, compared with only 8% of the urban-based samples. Respondents from both groups expressed strong interest in consuming more traditional foods, with 76% of urban-based respondents and 52% of reserve-based respondents indicating that they would prefer to consume traditional foods more frequently.

## Discussion

### Geographic and social dimensions of place and Indigenous food security

Research has shown that there are strong and dynamic relationships between financial insecurity, time scarcity, and food consumption (49). In our study, time was the dominant barrier to food security for reserve-based participants. Indeed, time scarcity is linked to the increased consumption of convenience foods (50), high rates of obesity and diet-related chronic diseases (47), and the decreased importance of homemade family meals (51). For urban participants, money was the dominant barrier to food security, and it is worth recalling that lower-income households are persistently more vulnerable to food insecurity and more likely to rely heavily on energy-dense, nutrient-poor, and more easily accessible convenience foods (52). Given these trends, it is not surprising that large numbers of both urban- and reserve-based First Nations individuals in southern Ontario are food insecure. Both groups have high proportions of lower-income households and are led by single mothers who are reportedly overreliant on convenience stores and their associated highly processed foods. Although the dominant barriers to consistently accessing healthy foods—time and money—are slightly different in each community, both pose risks for healthy diets.

While the survey data collected reflect broad similarities in experiences of food insecurity between on- and off-reserve participants, the place people lived in played the most important role in participants’ abilities to acquire food. Compared with the reserve-based participants, urban participants were not only more food insecure, they were also more likely to rely on soup kitchens and food banks, and to express desire to consume traditional foods more frequently. Regional studies on Indigenous food insecurity have illustrated how geographic distance and disconnection from family and one's home community can influence the dietary erosion of traditional foods for urban Indigenous people (52). It is also known that social supports and inclusion tend to help buffer against household food insecurity (15), but that food-insecure households are more likely to lack these social networks and resources (53). Our research suggests that the different experiences of food insecurity between on- and off-reserve participants are mediated in important ways by the material and cultural resources available to individuals within their informal social supports and institutional networks. In the reserve-based sample, in particular, these resources can act in protective ways against food insecurity. On-reserve, households are more likely to be led by 2 parents (29%), families tend to live nearer to one another, and there are often strong cultural food practices, such as community freezers and food boxes, gardens, and food sharing, which strengthen social cohesion and provide a social safety net. In this case, it is both the geographic and social aspects of place—fostered through family and community ties—that may improve access to traditional and locally acquired foods. For urban-based First Nations people, access to social and kinship networks are often more restricted as a result of their spatial, cultural, and social distance from the reserve. This access to social and kinship networks may be exacerbated by the fact that the urban-based households are predominantly led by single mothers (54%) who may face greater time and financial constraints.

Among Indigenous people living in both urban and reserve contexts, access to traditional foods is eroding over time and unprecedented dietary changes are occurring. Processes of environmental dispossession, occurring at both local and global scales, have restricted access to traditional lands, water, and food resources to such an extent that there has been a marked erosion in physical access to traditional foods in Indigenous diets. Indigenous communities have undergone unparalleled experiences of political and cultural violence. Operating largely through the Indian Act (1876), Indigenous people continue to be regulated by the only race-based legislation globally. While the assimilationist and colonial agenda that led to the development of the Indian Act has been revised many times, its legacy continues to harm traditional ways and livelihoods of Indigenous people in the contemporary context. The health consequences of environmental dispossession have occurred almost exclusively through disrupted food systems. In practical terms, this means that traditional food systems are increasingly being replaced by market foods. The associated nutrition transition of dietary change has been so profound that Indigenous diets in southwestern Ontario—both on- and off-reserve—are principally sourced by market foods purchased from grocery and convenience stores, and very minimally from traditional foods. Multiple studies have shown that traditional foods tend to be healthier and more nutrient dense than commercial foods (22, 24, 27) and that the vast proportion of food stock in grocery and convenience stores is made up of highly processed foods that are high in fats, sugars, and salts and low in micronutrients (53). Meals purchased at grocery and convenience stores are more likely to be higher in total calories, saturated and *trans* fats, and sodium and to include fewer servings of fruits, vegetables, and whole grains than food prepared at home (54). In addition, scholars have shown that convenience stores are more likely to be located in lower-income neighborhoods or food deserts, and that close proximity (<1 km) to convenience stores decreases the resident's likelihood of healthy eating and increases the risk of obesity (55). Convenience stores are also far more expensive than grocery stores. For example, in London, Ontario, identical food items cost an average of 1.6 times more at convenience stores compared with supermarkets, and residents of low-income neighborhoods have very limited access to grocery stores (56). These trends represent significant barriers for lower-income Indigenous families whose traditional foods are increasingly difficult to access, and for whom convenience foods are increasingly available. In our study, both urban- and reserve-based participants were heavily dependent on convenience and grocery stores, and both reserve- and urban-based families expressed a strong desire to consume more traditional foods. This suggests that these food environments are not only increasing the risk of First Nations people to obesity- and diet-related diseases but are providing foods that are incompatible with cultural preferences.

One of the motivations for the Food Choice Study was to assess and compare the dietary practices of urban- and reserve-based SOAHAC clients in relation to the Canada's Food Guide food-based recommendations, and the dietary practices of the Canadian population at large. In Canada, there has been a national trend toward healthier eating over the past 2 decades, including higher consumption (and greater diversity) of vegetables and fruits and lower consumption of red meats and other sources of saturated fats. Canada's updated Food Guide reflects this transition by recommending increasing consumption of whole grains, fruits, and vegetables as well as plant-based sources of protein. But these trends in healthy dietary components are not necessarily accessible, or affordable, for lower-income Indigenous populations (57). Our study shows insufficient consumption of both meat and milk and alternatives categories, low fruit and vegetable intake, and an overreliance on highly processed foods such as salty snacks, sweets, and soft drinks among both reserve- and urban-based participants. While there were some important differences in dietary patterns and food insecurity between sample groups, with urban Indigenous participants more likely to experience food insecurity, the common experiences of Indigeneity, dispossession, and marginalization are the driving force of Indigenous food insecurity in the greater London area. Further, while our sample was overrepresented by women, it must be emphasized that First Nations communities in southwestern Ontario, irrespective of gender or geographic location, are at considerable risk of food insecurity. Indigenous food environments are shaping dietary overreliance on processed foods of lower nutrient density that go against the former and current national food policy recommendations. Processes of environmental dispossession have made it extremely challenging to access healthier and culturally preferred traditional foods.

### Conclusions

As we consider the implications of this study, and possibilities for improving access to healthy, culturally appropriate diets for Indigenous people in both reserve and urban contexts, we follow the direction of Cidro et al. (41), Daigle (58), and Delormier et al. (59), who promote the concept of Indigenous Food Sovereignty (IFS). In the broadest sense, IFS implies a central commitment for communities to make their own decisions about their food systems, and it relies on a number of important concepts, including sharing practices, self-determination, and decolonization (34).

In our understanding, the uptake of IFS and its related concepts necessitate a return to the cultural values and ways of knowing about food that has been interrupted in the past hundred years, which is through processes of environmental repossession. In its widest framing, environmental repossession refers to the social, cultural, and political processes by which Indigenous peoples and communities are reclaiming their traditional lands and ways of life (60). The concept of environmental repossession is rooted in the idea that Indigenous peoples’ health, ways of living, and Indigenous knowledge systems are highly dependent on access to their traditional lands and territories. While the unprecedented urbanization of Indigenous people has been characterized as paradoxical to traditional ways of knowing and understanding, we challenge this assumption by sharing an example wherein urban organizations and communities have supported principles key for IFS, and with positive implications. In Vancouver, British Columbia, the Urban Aboriginal Kitchen Garden (61) is a project that brought Indigenous people from Vancouver's downtown east-side, one of the poorest and most disenfranchised neighborhoods in the city, to the University of British Columbia Farm to teach them how to garden. Over the course of several weeks and months, food is tended to, and grown, then harvested and eaten together. Alongside the important social relationships this garden created for people who tended it, the garden also provided an important place for the resurgence of Indigenous knowledge. The garden created an intentional space for people to re-engage with cultural practices, knowledge, and a whole food system from which they had been disconnected.

The research reported in this study illustrates that, in the reserve context, there are multiple ways in which social supports positively mediate the relationship between income and food security. This represents an important area of hope and future investigation. Indigenous communities in southwestern Ontario are collaborating to provide for each other, and to continue to create dignified food systems that not only improve food security but also restore long-held cultural and social values, knowledges, and practices that have historically maintained community health. Scholars, activists, and community organizations who work to improve Indigenous food security in the off-reserve context must keep this in mind and find ways to both acknowledge and support the critically important work of urban Indigenous organizations, such as the Southwest Ontario Aboriginal Health Access Centre and its affiliated centers across Ontario. In Canadian urban centers, many of these organizations are chronically underresourced and often regulated by the larger provincial programs through which they are fiscally funded (62). Despite the strict funding rules and political pressures endured by urban Indigenous organizations, their willingness to engage in research, and to search for solutions that will not only improve health outcomes for their clients but also support cultural resurgence, is a shining example of what is possible.

In this project, we engaged in an interdisciplinary framing of Indigenous food security that sought to understand it and its determinants in a way that was congruent with Indigenous knowledges and place- and land-based ways of understanding and experiencing food and food security (31, 34). Our partnership with SOAHAC was critically important, as it was they who steered this research from its early development through to the data analysis and dissemination. SOAHAC recognized a need for a comprehensive evaluation of broader constraints that influenced food choices among their clientele, and entered this research collaboration with a goal to use the findings in a way that could inform more appropriate programming, especially for Indigenous women. Moving forward, we urge those working in food and nutritional sciences to consider such collaborative and interdisciplinary approaches in their studies of food security. The pathway toward IFS contains important cultural, social, and political dimensions that are fundamentally place-based (58, 59), and will become knowable through integrative, collaborative research programs that places Indigenous peoples, their concerns and their knowledges firmly at the center of the research.

## Notes

This research received funding from an Aboriginal Pilot Project of the Heart and Stroke Foundation of Ontario.

Author disclosures: The authors report no conflicts of interest.

The Heart and Stroke Foundation played no role in the design, implementation, analysis, or interpretation of the study.

Abbreviations used: FFQ, food-frequency questionnaire; HFIAS, Household Food Insecurity Access Scale; IFS, Indigenous Food Sovereignty; SOAHAC, Southwest Ontario Aboriginal Health Access Centre.
